# Zfhx3 is required for proper spindle assembly and chromosome segregation during oocyte meiosis I in mice

**DOI:** 10.3389/fcell.2026.1811072

**Published:** 2026-03-27

**Authors:** Chenyang Huang, Haoya Chang, Xin Zhao, Siyuan Chen, Xiaohong Wang, Jin-Tang Dong

**Affiliations:** 1 Department of Human Cell Biology and Genetics, SUSTech Homeostatic Medicine Institute, School of Medicine, Southern University of Science and Technology, Shenzhen, China; 2 Department of Obstetrics and Gynecology, Tangdu Hospital, Air Force Medical University, Xi’an, China

**Keywords:** aneuploidy, chromosome segregation, meiosis progression, mouse oocyte, spindle assembly, Zfhx3

## Abstract

**Background:**

ZFHX3, a multifunctional transcription factor, plays pivotal roles in a variety of physiological and pathological processes, including neuronal differentiation, development, atrial fibrillation, and cancer. Notably, homozygous deletion of Zfhx3 is embryonic lethal, whereas its hemizygous deletion results in reduced neonatal body weight and increased preweaning mortality in mice. Also, a deletion mutation in Zfhx3 is significantly associated with reduced litter size, a key reproduction-related trait in goats, suggesting that ZFHX3 is involved in reproductive development. However, its specific function in female reproduction remains unclear. Given that oocyte meiosis is a fundamental process in female reproduction, we investigated the role of Zfhx3 in this process.

**Method:**

We performed siRNA microinjection, immunofluorescence staining, chromosome spreading, Western blotting and other experiments to investigate the function of Zfhx3 in mouse oocyte meiosis.

**Results:**

We found that Zfhx3 was present in both the nucleus and cytoplasm in GV oocytes and became predominantly localized to the cytoplasm after germinal vesicle breakdown. Knockdown of Zfhx3 caused failure of first polar body extrusion due to sustained activation of the spindle assembly checkpoint (SAC). Zfhx3-deficient oocytes were defective in spindle assembly, microtubule-kinetochore attachment, and chromosome segregation during meiosis I, resulting in aneuploidy in MII oocytes. These defects could be ameliorated by ectopic expression of *Zfhx3* mRNA.

**Conclusion:**

Our findings provide evidence for an essential role of Zfhx3 in spindle assembly and chromosome segregation during mouse oocyte meiosis I, and provide a mechanistic basis for its mutations in female reproductive disorders.

## 1 Introduction

Meiosis is a fundamental process in oogenesis (He et al., 2021). Successful meiotic maturation involves two consecutive asymmetric oocyte divisions, which are essential for healthy embryo development and the transmission of genetic material (Telfer et al., 2023; Giaccari et al., 2024). Disruption of this process can result in spindle abnormalities and error-prone chromosome segregation, thereby elevating the risk of aneuploidy and defective embryo development (Charalambous and Schuh, 2023; So et al., 2022; Zhu et al., 2025a). Therefore, a thorough understanding of the molecular mechanisms of meiotic progression in oocytes is crucial for female reproductive health.

Spindle assembly is critical for oocyte meiosis. The oocyte meiotic spindle assembles in the absence of centrosomes, the main microtubule-organizing centers in somatic cells, which relies on acentriolar microtubule-organizing centers (aMTOCs). The assembly of the meiotic spindle begins with microtubule nucleation at aMTOCs (Mogessie and Schuh, 2018). The mechanism underlying this process is highly specialized. In mouse oocytes, nucleation occurs primarily at cytoplasmic aMTOCs (Schuh and Ellenberg, 2007), whereas in human oocytes, kinetochores serve as the major sites (Wu et al., 2022). Following nucleation, microtubules first organize into an apolar, ball-like structure, which subsequently undergoes a stereotypical transformation into an elongated, barrel-shaped bipolar spindle (Mogessie and Schuh, 2018; Mullen and Wignall, 2019).

During oocyte meiosis, the spindle assembly checkpoint (SAC) safeguards against premature anaphase onset by inhibiting the anaphase-promoting complex/cyclosome (APC/C), thereby ensuring precise chromosome segregation (Marston and Wassmann, 2017). BubR1 and Bub3, key SAC components, are critical for the surveillance of kinetochore-microtubule attachment (Li et al., 2009; Wei et al., 2010). Proper chromosome alignment and the establishment of stable kinetochore-microtubule connections silence the SAC, which, in turn, triggers APC/C activation, leading to chromosome segregation and anaphase onset (McGuinness et al., 2009). The SAC in oocytes is less stringent than in somatic cells due to their large cytoplasmic volume (Lane and Jones, 2017; Kyogoku and Kitajima, 2017), leading to a higher incidence of errors in chromosome alignment and segregation, and thereby contributing to the elevated rate of aneuploidy in oocytes.

ZFHX3 plays pivotal roles in diverse physiological and pathological processes, including neuronal differentiation (Baca et al., 2024), circadian regulation (Parsons et al., 2015), atrial fibrillation (Hwang et al., 2021), and cancer (Fu et al., 2020; Hu et al., 2019; Dong et al., 2020). Homozygous deletion of Zfhx3 is embryonic lethal, whereas its hemizygous deletion results in reduced neonatal body weight and increased preweaning mortality in mice, indicating a role of ZFHX3 in development (Sun et al., 2012). A deletion mutation in Zfhx3 is also significantly associated with reduced litter size, a key reproduction-related trait in goats (Wang K et al., 2020), suggesting a role for ZFHX3 in regulating female reproduction. However, whether ZFHX3 modulates oocyte meiosis has not been directly examined.

In the present study, we investigated the function of Zfhx3 in oocyte meiotic maturation using knockdown and rescue approaches. We discovered that Zfhx3 is essential for ensuring precise spindle assembly, microtubule-kinetochore attachment, and chromosome segregation during mouse meiosis I, thereby safeguarding oocyte euploidy.

## 2 Materials and methods

### 2.1 Animals

Six-week-old female ICR strain mice were obtained from Beijing Weitonglihua Experimental Animal Technology Co., Ltd. and maintained on a 12-h dark/12-h light cycle. Food and sterilized water were provided *ad libitum*. Mice were used for experiments after a 1-week acclimatization period. All experiments were approved by the Institutional Animal Care and Use Committee of Southern University of Science and Technology (Approval identification: SUSTech-JY202202013).

### 2.2 Oocyte collection and culture

As previously described (Chang et al., 2024a), GV oocytes were collected from female mice following superovulation. Briefly, superovulation was induced by intraperitoneal administration of 10 IU pregnant mare serum gonadotropin (PMSG; Ningbo Hormone Products Co.). After 46–48 h, GV oocytes were harvested from the ovaries and placed into M2 medium supplemented with 200 μM three-isobutyl-1-methylxanthine (IBMX) (HY-12318, MCE) to maintain meiotic arrest. Only oocytes with normal morphology were selected and subsequently cultured for *in vitro* maturation in IBMX-free M2 medium.

### 2.3 Real-time quantitative PCR

Real-time quantitative PCR was carried out according to the established protocol (Chang et al., 2024b), with minor modifications. Briefly, 40 oocytes were collected for each sample. After three washes with RNase-free PBS, samples were transferred into Cells-to-Signal lysis buffer (AM8728; Invitrogen). cDNA was synthesized using PrimeScript™ RT reagent Kit (RR047A; Takara). qRT-PCR was performed using Takara SYBR Premix Ex Taq II (RR820A; Takara) on CFX96 RT-PCR System (Bio-Rad). The nucleotide sequences of the primers are: for *Zfhx3* 5′-aga​gca​aga​ggg​cag​cgt​cat​c-3’ (forward) and 5′-cgg​ttc​acg​tca​gcg​ttg​cta​ta-3′ (reverse); for *Actin*, 5′-gtg​acg​ttg​aca​tcc​gta​aag​a-3’ (forward), 5′-gcc​gga​ctc​atc​gta​ctc​c-3’ (reverse). The PCR program was set as follows: initial denaturation at 95 °C for 30 s, followed by 40 cycles of 95 °C for 5 s and 60 °C for 30 s. *Actin* was used as an internal control for normalization.

### 2.4 Western blot analysis

200 oocytes for each sample were collected and transferred into 10 μL 2X SDS buffer (P0015L; Beyotime), and heated at 98 °C for 8 min. Proteins were separated by SDS-PAGE using 4% (for Zfhx3) and 10% (for beta Actin) gels and then transferred to PVDF membranes (Millipore) (Wu et al., 2020). The membranes were blocked using QuickBlock™ Blocking Buffer (P0222; Beyotime) for 20 min at room temperature (RT), and incubated overnight at 4 °C with Anti-ZFHX3 antibody (1:1000; PD010; MBL), HA antibody (1:1000; 3724; CST), or Anti-beta Actin antibody (1:1000; AC004; Abclonal). After three washes with PBST, samples were incubated at RT for 1 h with HRP-conjugated anti-rabbit/mouse IgG (H + L) antibodies (1:5000). Finally, membranes were visualized using the ECL Plus Western blotting Detection System (Tanon).

### 2.5 Immunofluorescence staining

Immunofluorescence staining was performed as previously described (Chang et al., 2024a). Oocytes at different stages were fixed in 4% paraformaldehyde for 40 min, permeabilized with PBS containing 0.5% Triton X-100 for 20 min, and then blocked in 1% BSA for 1 h at RT. Then, samples were incubated with primary antibody, including Anti-Alpha-Tubulin conjugated with Alexa 488 (1:1000; 322588; Thermo Fisher Scientific) and Anti-ZFHX3 antibody (1:1000; PD010; MBL) overnight at 4 °C. After three washes in PBS containing 0.1% TritonX-100, samples were then incubated with secondary antibodies, including anti-Mouse Alexa 488 (1:1000; A-11001; Thermo Fisher Scientific), anti-Rabbit Alexa 594 (1:1000; A-11002; Thermo Fisher Scientific) for 1 h at RT. Subsequently, the samples were mounted in an antifade mounting medium containing Hoechst 33,342 (P0133; Beyotime) for nuclear counterstaining and visualized using a Leica TCS SP8 confocal microscope.

### 2.6 Microinjection of siRNA and mRNA

Microinjection was carried out using an Eppendorf microinjection system. Zfhx3 siRNA sequence is 5′- ACG​UGA​CAC​GUU​CGG​AGA​A-3’. Control siRNA sequence is 5′- UUC​UCC​GAA​CGU​GUC​ACG​U-3’. 3–5 pL of siRNA (1 mM) was injected into GV oocytes. Zfhx3 cDNA was cloned into the pcDNA3.1 (+)-HA vector. The cRNAs were transcribed *in vitro* using the T7 mMessage mMachine *in vitro* transcription kit (AM1344, Thermo Fisher Scientific), followed by purification with the RNeasy kit (74,004; Qiagen). At 24 h after siRNA injection, GV oocytes were microinjected with HA-Zfhx3 mRNA (300 ng/μL) for rescue experiments.

### 2.7 Cold treatment

MI oocytes were subjected to cold treatment by incubation on ice for 10–15 min before immunofluorescence staining.

### 2.8 Chromosome spreading

Oocytes were subjected to chromosome spreading as previously described (Chang et al., 2024b). In brief, oocytes were placed in Tyrode’s solution (T1788, Sigma–Aldrich) to remove the zona pellucida, which were then incubated in a chromosome-spreading solution for 4–6 h at RT. After the samples were air-dried, the immunofluorescence staining procedure was performed according to the standard protocol outlined above. Primary antibodies: Anti-Centromere Protein Antibody (1:50; 15–234; Antibodies Incorporated), and anti-BUB3 mouse monoclonal antibody (1:50; 376,506; Santa Cruz Biotechnology). Secondary antibodies: Goat anti-Human IgG secondary antibody conjugated with Alexa Fluor 647 (1:1000; A-21445; Thermo Fisher Scientific) and Goat anti-Mouse IgG (H + L) secondary antibody conjugated with Alexa Fluor 488 (1:1000; A-11001; Thermo Fisher Scientific).

### 2.9 Statistical analysis

All experiments were performed with at least three independent biological replicates, wherein oocytes were newly collected from different batches of mice. Data are presented as the mean ± standard deviation (SD). Significance was determined using a two-tailed Student’s t-test for dual comparisons. Statistical significance was defined as *p* < 0.05. Statistical analyses were conducted using GraphPad Prism 9.5.

## 3 Results

### 3.1 Expression and localization of Zfhx3 during mouse oocyte meiosis

**FIGURE 1:**
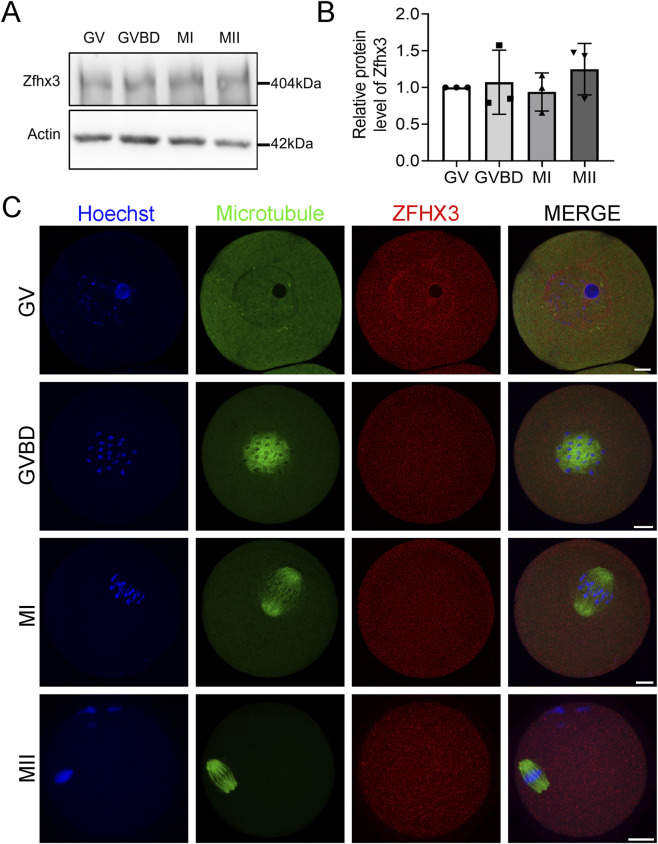
Expression and cellular localization of Zfhx3 during mouse oocyte meiosis. **(A)** Protein levels of Zfhx3 in oocytes at GV, GVBD, MI, and MII stages, as detected by Western blotting. GV, GVBD, MI, and MII oocytes were collected at 0 h, 3 h, 7h, and 14 h post-IBMX release, respectively. **(B)** The quantitative analysis of the Zfhx3 protein band intensities in GV, GVBD, MI, and MII oocytes from panel **(A)**. The bar height and error bars depict the mean and SD, respectively. **(C)** Representative immunofluorescence images show the subcellular localization of Zfhx3 in oocytes at GV (n = 27), GVBD (n = 19), MI (n = 12), and MII (n = 22). Scale bar, 20 μm.

The protein expression and subcellular localization of Zfhx3 were first analyzed at different developmental stages during mouse oocyte meiotic maturation. Western blotting demonstrated that Zfhx3 was persistently expressed from GV to MII stage during oocyte meiosis (Figures 1A,B). Immunofluorescence staining and confocal imaging were then performed to determine the subcellular localization of Zfhx3 during oocyte meiosis. Whereas Zfhx3 was present in both the nucleus and cytoplasm of GV oocytes, it was accumulated predominantly in the cytoplasm of GVBD, MI, and MII oocytes (Figure 1C). Interestingly, Zfhx3 clearly exhibited nuclear localization in surrounded nucleolus (SN) oocytes, but was not detected in non-surrounded nucleolus (NSN) oocytes (Supplementary Figure S1).

### 3.2 Zfhx3 depletion impairs first polar body extrusion during oocyte meiosis

**FIGURE 2:**
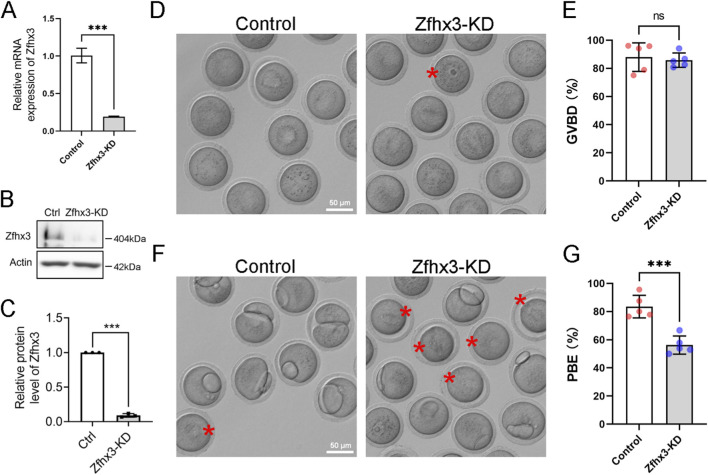
*Zfhx3* knockdown impairs first polar body extrusion during mouse oocyte meiosis. **(A)** Zfhx3 mRNA levels in control and Zfhx3-KD oocytes, as determined by RT-qPCR. **(B)** Protein levels of Zfhx3 in control and Zfhx3-KD oocytes, as determined by Western blotting. **(C)** The quantitative analysis of Zfhx3 protein band intensities in control and Zfhx3-KD oocytes from panel **(B) (D,E)** Representative images of GVBD in control and Zfhx3-KD oocytes **(D)** and the bar graph of GVBD ratios of control (n = 97) and Zfhx3-KD (n = 101) oocytes **(E)**. Asterisks indicate oocytes that failed to undergo germinal vesicle breakdown. **(F,G)** Representative images of the first polar body extrusion (PBE) in control and Zfhx3-KD oocytes **(F)** and the bar graph of the PBE ratios of control (n = 88) and Zfhx3-KD (n = 86) oocytes **(G)**. Asterisks indicate oocytes that failed to undergo the extrusion of the first polar body. The bar height and error bars depict the mean and SD, respectively. ***, *p* < 0.001. *P*-values were calculated using a two-tailed unpaired Student’s t-test.

We next investigated the effects of *Zfhx3* knockdown on mouse oocyte meiosis. As demonstrated by qPCR, *Zfhx3* mRNA level was significantly decreased after Zfhx3-siRNA injection (Figure 2A; *p* < 0.001). Western blotting showed that the Zfhx3 protein level in the Zfhx3-KD group was markedly lower than that in the control group (Figures 2B,C; *p* < 0.001). Oocyte meiosis during *in vitro* maturation was determined by the incidence of GVBD at 3 h and PBE at 14 h. As shown in Figures 2D,E, *Zfhx3* silencing did not affect the GVBD rates of mouse oocytes (Control: 87.98% ± 10.17%, n = 97; Zfhx3-KD: 85.83% ± 5.15%, n = 101; *p* > 0.05). However, *Zfhx3* silencing led to a decrease in PBE rates compared to oocytes from the control group (Control: 83.48% ± 8.03%, n = 88; Zfhx3-KD: 56.27% ± 6.46%, n = 86; *p* < 0.001) (Figures 2F,G). Collectively, these data highlighted the necessity of Zfhx3 for mouse oocyte meiotic maturation.

### 3.3 Zfhx3 depletion impairs spindle assembly and chromosome alignment in MI oocytes

**FIGURE 3:**
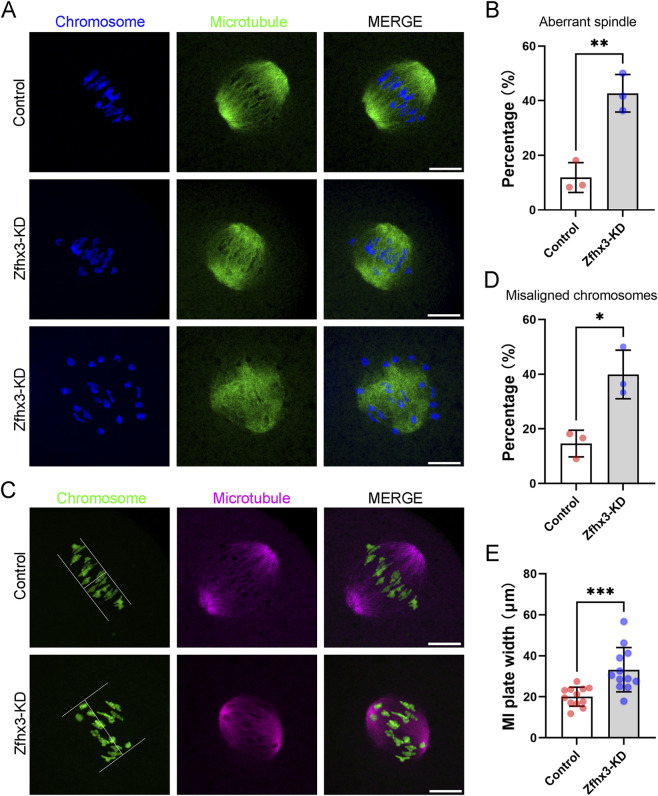
Zfhx3 knockdown disturbs spindle formation and chromosome alignment in MI oocytes. **(A)** Typical images of spindle morphology in MI oocytes from the control and Zfhx3-KD groups. MI oocytes were collected following a 7 h culture in IBMX-free M2 medium. Scale bar, 10 μm. **(B)** Bar graph comparing the percentages of aberrant spindles in control (n = 34) and Zfhx3-KD (n = 35) oocytes. Individual data points correspond to independent experiments. The bar height and error bars depict the mean and SD, respectively. **(C)** Typical images of chromosome alignment in MI oocytes from the control and Zfhx3-KD groups. **(D)** Bar graph comparing the percentages of misaligned chromosomes in control (n = 34) and Zfhx3-KD (n = 35) oocytes. Individual dots correspond to independent experiments. The bar height and error bars depict the mean and SD, respectively. **(E)** Bar graph comparing MI plate width in oocytes from the control (n = 12) and Zfhx3-KD (n = 12) groups. Each data point represents an individual oocyte. The bar height and error bars depict the mean and SD, respectively. *, *p* < 0.05; **, *p* < 0.01; ***, *p* < 0.001. *P*-values were calculated using a two-tailed unpaired Student’s t-test.

To better understand the cellular mechanisms underlying the failure in polar body extrusion upon Zfhx3 knockdown, we then examined spindle assembly and chromosome alignment in MI oocytes by immunofluorescence staining. As shown in Figures 3A,B, Zfhx3 knockdown significantly increased the percentage of aberrant spindle morphology in MI oocytes (Control: 11.87% ± 5.48%, n = 34; Zfhx3-KD: 42.68% ± 6.88%, n = 35; *p* < 0.01). Zfhx3 depletion also resulted in misaligned chromosomes in MI oocytes (Control: 14.65% ± 4.87%, n = 34; Zfhx3-KD: 39.90% ± 8.88%, n = 35; *p* < 0.05) (Figures 3C,D), with increased MI plate width (Control: 20.06 ± 4.65 μm, n = 12; Zfhx3-KD: 33.23 ± 10.77 μm, n = 12; *p* < 0.001) (Figure 3E). These findings indicate that Zfhx3 is essential for spindle assembly and chromosome alignment during oocyte meiosis I.

### 3.4 Zfhx3 knockdown induces defective K-M attachment and aneuploidy in oocytes

**FIGURE 4:**
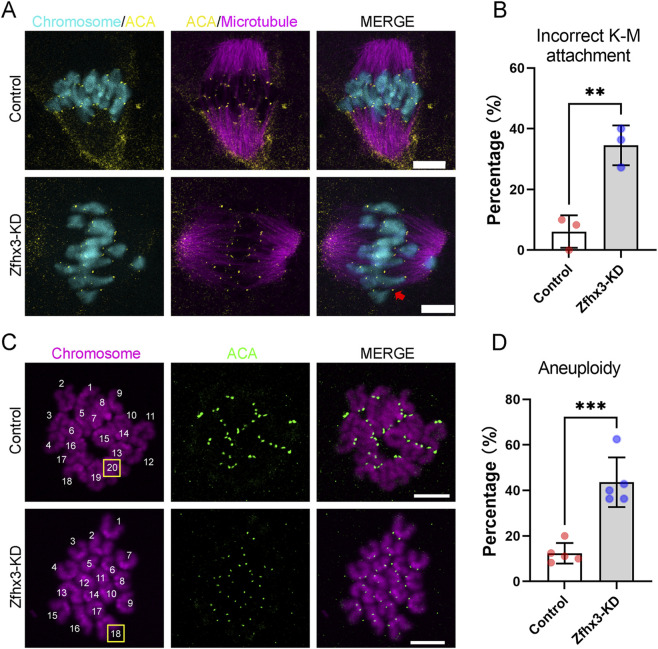
Zfhx3 knockdown affects K-M attachment and induces aneuploidy in oocytes. **(A)** Typical images of the K-M attachment in control and Zfhx3-KD oocytes at the MI stage. Scale bar, 10 μm. MI oocytes were collected following a 7 h culture in IBMX-free M2 medium. The arrow denotes incorrect kinetochore-microtubule attachment. **(B)** Bar graph comparing the percentages of incorrect K-M attachment in control (n = 34) and Zfhx3-KD (n = 32) oocytes. Individual data points correspond to independent experiments. The bar height and error bars depict the mean and SD, respectively. **(C)** Typical images of euploid and aneuploid oocytes in the control and Zfhx3-KD groups. Scale bar, 10 μm. **(D)** Bar graph comparing the percentages of aneuploidy in oocytes from control (n = 44) and Zfhx3-KD (n = 47) groups. MII oocytes were collected following a 14 h culture in IBMX-free M2 medium. Individual data points correspond to independent experiments. The bar height and error bars depict the mean and SD, respectively. **, *p* < 0.01; ***, *p* < 0.001. *P*-values were calculated using a two-tailed unpaired Student’s t-test.

Given that abnormal spindle assembly and chromosome alignment are highly correlated with defective K-M attachment and cause aneuploidy in oocytes, we then examined the effects of *Zfhx3* depletion on K-M attachment and aneuploidy. As expected, the incidence of abnormal K-M attachment in Zfhx3-KD oocytes was higher than that of the control oocytes (Control: 6.11% ± 5.36%, n = 34; Zfhx3-KD: 34.54% ± 6.56%, n = 32; *p* < 0.01) (Figures 4A,B). Zfhx3 depletion also led to a significant increase in the incidence of aneuploidy in MII oocytes (Control: 12.39% ± 4.52%, n = 44; Zfhx3-KD: 43.62% ± 10.90%, n = 47; *p* < 0.001) (Figures 4C,D).

### 3.5 Zfhx3 depletion compromises SAC release during meiosis I

**FIGURE 5:**
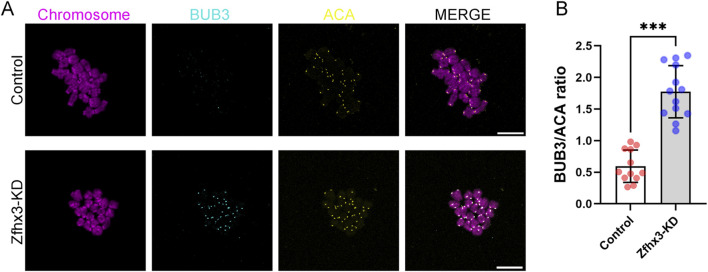
Zfhx3 knockdown disturbs SAC release during oocyte meiosis I. **(A)** Representative images of BUB3 and ACA staining signals in control and Zfhx3-KD oocytes at 9 h after release from IBMX. Scale bar, 10 μm. **(B)** The ratios of BUB3 to ACA were determined in control (n = 12) and Zfhx3-KD (n = 13) oocytes. Each data point represents an individual oocyte. The bar height and error bars depict the mean and SD, respectively. ***p < 0.001. *P*-values were calculated using a two-tailed unpaired Student’s t-test.

We subsequently examined the activity of BUB3, a key spindle assembly checkpoint (SAC) protein, at centromeres in oocytes following a 9 h culture in IBMX-free M2 medium. Immunofluorescence staining demonstrated that the ratio of BUB3 to ACA in Zfhx3-deficient oocytes was significantly higher than that in control oocytes (Figures 5A,B; *p* < 0.001), suggesting that Zfhx3 knockdown disrupts SAC release during meiosis I.

### 3.6 Ectopic expression of Zfhx3 ameliorates meiotic defects in Zfhx3-deficient oocytes

**FIGURE 6:**
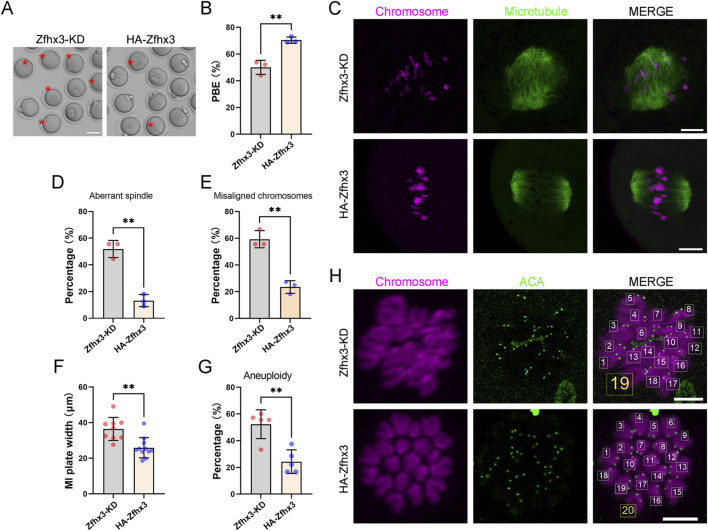
Ectopic expression of HA-Zfhx3 mRNA ameliorates meiotic defects in Zfhx3-deficient oocytes. **(A)** Representative images of the first polar body extrusion (PBE) in oocytes from Zfhx3-KD and HA-Zfhx3 groups. Scale bar, 50 μm. Asterisks indicate oocytes that failed to undergo the extrusion of the first polar body. **(B)** Bar graph comparing the PBE ratios of oocytes from Zfhx3-KD (n = 74) and HA-Zfhx3 (n = 81) groups. Individual data points correspond to independent experiments. **(C)** Typical images of spindle morphology and chromosome alignment in oocytes from Zfhx3-KD and HA-Zfhx3 groups. Scale bar, 10 μm. **(D,E)** Quantification of the percentages of oocytes with aberrant spindles and misaligned chromosomes in Zfhx3-KD (n = 27) and HA-Zfhx3 (n = 30) groups. Individual data points correspond to independent experiments. **(F)** Measurement of the width of the MI plate in oocytes from Zfhx3-KD and HA-Zfhx3 groups. Each data point in the graph represents an individual oocyte. **(G)** Quantification of the percentages of aneuploidy in oocytes from Zfhx3-KD (n = 44) and HA-Zfhx3 (n = 36) groups. Individual data points correspond to independent experiments. **(H)** Representative images of chromosome spreads in MII oocytes from Zfhx3-KD (n = 44) and HA-Zfhx3 (n = 36) groups. Scale bar, 10 μm **, *p* < 0.01. *P*-values were calculated using a two-tailed unpaired Student’s t-test.

To rule out off-target effects of siRNA against *Zfhx3*, we ectopically expressed HA-tagged Zfhx3 (HA-Zfhx3) in Zfhx3-deficient oocytes. Then we examined key meiosis features, including first polar body extrusion, spindle assembly, chromosome alignment, and aneuploidy in both Zfhx3-deficient and HA-Zfhx3-expressing oocytes. At 4 h after microinjection, the expression of HA-Zfhx3 protein in oocytes was confirmed by Western blotting (Supplementary Figure S2A). Ectopic expression of HA-Zfhx3 significantly increased the PBE rate compared with the Zfhx3-KD oocytes (Zfhx3-KD: 50.01% ± 5.27%, n = 74; HA-Zfhx3: 70.43% ± 2.30%, n = 81; *p* < 0.01) (Figures 6A,B). There was no significant difference in the GVBD rates between Zfhx3-KD and HA-Zfhx3 oocytes (Supplementary Figure S2B; *p* > 0.05). The following parameters were also significantly altered by HA-Zfhx3 re-expression: the percentages of abnormal spindle (Zfhx3-KD: 51.85% ± 6.42%, n = 27; HA-Zfhx3: 13.26% ± 4.59%, n = 30; *p* < 0.01) (Figures 6C,D); misaligned chromosomes (Zfhx3-KD: 59.26% ± 6.41%, n = 27; HA-Zfhx3: 23.48% ± 4.73%, n = 30; *p* < 0.01) (Figure 6E); aneuploidy (Zfhx3-KD: 52.32% ± 10.77%, n = 44; HA-Zfhx3: 24.33% ± 8.85%, n = 36; *p* < 0.01) (Figures 6G,H); and MI plate width (Zfhx3-KD: 36.44 ± 6.42 μm, n = 9; HA-Zfhx3: 25.90 ± 5.70 μm, n = 11; *p* < 0.01) (Figure 6F). Collectively, these results demonstrated that Zfhx3 plays a critical role during mouse oocyte meiosis I.

## 4 Discussion

**FIGURE 7:**
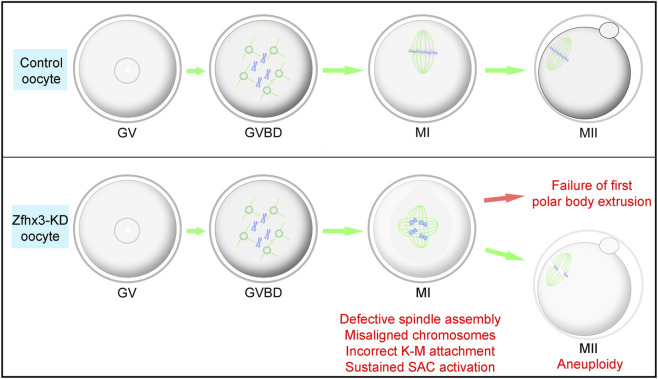
Roles of Zfhx3 in mouse oocyte meiosis. Zfhx3 knockdown causes the failure of the first polar body extrusion due to sustained activation of SAC, resulting in defects in oocyte spindle assembly, microtubule‐kinetochore attachment, and chromosome alignment during meiosis I, as well as subsequent aneuploidy in MII oocytes.

ZFHX3 plays pivotal roles in diverse physiological and pathological processes, including neuronal differentiation (Baca et al., 2024), circadian regulation (Parsons et al., 2015), atrial fibrillation (Hwang et al., 2021), and cancer (Fu et al., 2020; Hu et al., 2019; Dong et al., 2020). In this study, we performed siRNA microinjection, immunofluorescence staining, and chromosome spreading, among other methods, to investigate the function of Zfhx3 in mouse oocyte meiosis. Our findings demonstrated that Zfhx3 is essential for ensuring precise spindle assembly, K-M attachment, and chromosome segregation during meiosis I in mice, thereby safeguarding oocyte euploidy (Figure 7).

Zfhx3 is primarily localized to the nucleus in mouse GV oocytes, which is similar to its subcellular localization in breast epithelial cells at interphase (Li et al., 2017). After nuclear envelope breakdown, ZFHX3 remains mainly concentrated at chromosomes in breast epithelial cells (Li et al., 2017), whereas our current study indicates a predominant localization to the cytoplasm in GVBD, MI, and MII oocytes. The different distribution patterns imply that Zfhx3 may play distinct roles in mitosis and meiosis. Interestingly, Zfhx3 exhibits a clear nuclear localization in SN oocytes but not in NSN oocytes. Dynamic changes in chromatin accessibility, along with H3K4me3, orchestrate the transcriptional reprogramming that drives oocyte NSN-to-SN transition (Zhu et al., 2025b). ZFHX3 has been reported to regulate chromatin accessibility and histone methylation H3K4me3 (Bafna et al., 2025), suggesting a possible participation in the NSN-to-SN transition in oocytes. In the future, it will be beneficial to investigate whether Zfhx3 is involved in NSN-to-SN transition using oocyte-specific knockout mice.

Our findings revealed that the transcription factor Zfhx3 is required for spindle assembly and chromosome alignment during mouse oocyte meiosis I, a function distinct from its role in mitosis. Given the transcriptionally quiescent state in oocytes during meiotic division, the function of transcription factors in spindle assembly and chromosome alignment during oocyte meiosis remains relatively unexplored. It has been demonstrated that the transcription factor Mcrs1 is essential for spindle organization during meiosis I (Ju et al., 2023), consistent with our findings. Another transcription factor, Tfap2a, has also been reported to regulate spindle assembly and chromosome alignment in mouse oocytes (Lin et al., 2022). Taken together with the fact that oocytes are transcriptionally quiescent during meiotic maturation, regulation of spindle assembly and chromosome segregation during oocyte meiosis I by Zfhx3 is considered transcription-independent. The mechanisms underlying such a regulation require further investigation.

The proper attachment of the kinetochore to the microtubule is a fundamental prerequisite for achieving bipolar chromosome alignment (Maiato and Silva, 2023). As expected, we observed that Zfhx3 depletion compromises the stability of kinetochore-microtubule attachment. The spindle assembly checkpoint (SAC) monitors the attachment status between the kinetochore and the microtubule. Incorrect kinetochore-microtubule attachment promotes continuous recruitment and activation of the SAC, which may cause the failure of the metaphase I-to-anaphase I transition during oocyte meiosis. BUB3, a core component of the SAC, is released from kinetochores in oocytes at the late MI stage (Li et al., 2009). Thus, we examined the BUB3 signal in oocytes after 9 h of culture. The results indicated Zfhx3-deficient oocytes exhibited sustained BUB3 activation at kinetochores. This finding may explain the failure of first polar body extrusion caused by Zfhx3 knockdown. Zfhx3 induces cell-cycle arrest coupled to neuronal differentiation in the developing rat brain (Jung et al., 2005). These findings establish a role for Zfhx3 in cell cycle regulation during both mitosis and meiosis.

While misalignment of chromosomes triggered SAC-mediated arrest at MI, aneuploidy was also detected in Zfhx3-deficient oocytes that subsequently reached MII. Meiotic progression in oocytes is often tolerant of limited chromosome misalignment, reflecting a reduced stringency of the SAC for defective chromosome alignment compared to somatic cells (Kyogoku and Kitajima, 2017; Lane and Jones, 2017). Numerous studies have confirmed this view. KLF15 depletion leads to defective K-M attachment and subsequent SAC activation, but a proportion of KLF15-depleted oocytes escape SAC surveillance, producing aneuploid oocytes (Zou et al., 2022). Wang et al. also found that WDR62 knockdown induces incorrect K-M attachment and activates the SAC in MI oocytes, leading to aneuploid generation (Wang Y et al., 2020), consistent with our findings.

In conclusion, we report that Zfhx3 ensures the proper K-M attachment, spindle assembly, and chromosome segregation during mouse meiosis I, thereby safeguarding oocyte euploidy. Our findings uncover a unique role for Zfhx3 during mouse oocyte meiosis.

## Data Availability

The original contributions presented in the study are included in the article/Supplementary Material, and further inquiries can be directed to the corresponding authors.
