# Supplementary material for: Zfhx3 is required for proper spindle assembly and chromosome segregation during oocyte meiosis I in mice
Source: Front Cell Dev Biol. 2026 Mar 27;14:1811072. doi: 10.3389/fcell.2026.1811072 (PMC13066153; doi:10.3389/fcell.2026.1811072)
Supplement: Supplementary file 1 [file DataSheet1.docx]

Supplementary Material

Zfhx3 is Required for Proper Spindle Assembly and Chromosome Segregation during Oocyte Meiosis I in Mice

Chenyang Huang ^1^, Haoya Chang ^2^, Xin Zhao ^1^, Siyuan Chen ^1^, Xiaohong Wang ^2,^ * and Jin-Tang Dong ^1,^ *

^1^ Department of Human Cell Biology and Genetics, SUSTech Homeostatic Medicine Institute, School of Medicine, Southern University of Science and Technology, Shenzhen, China

^2^Department of Obstetrics and Gynecology, Tangdu Hospital, Air Force Medical University, Xi’an, China

*** Correspondence:**wangxh919@fmmu.edu.cn (X.H.W.); dongjt@sustech.edu.cn (J.T.D.)

Keywords: Zfhx3; mouse oocyte; meiosis progression; spindle assembly; chromosome segregation; aneuploidy

# Supplementary Figure 1

**
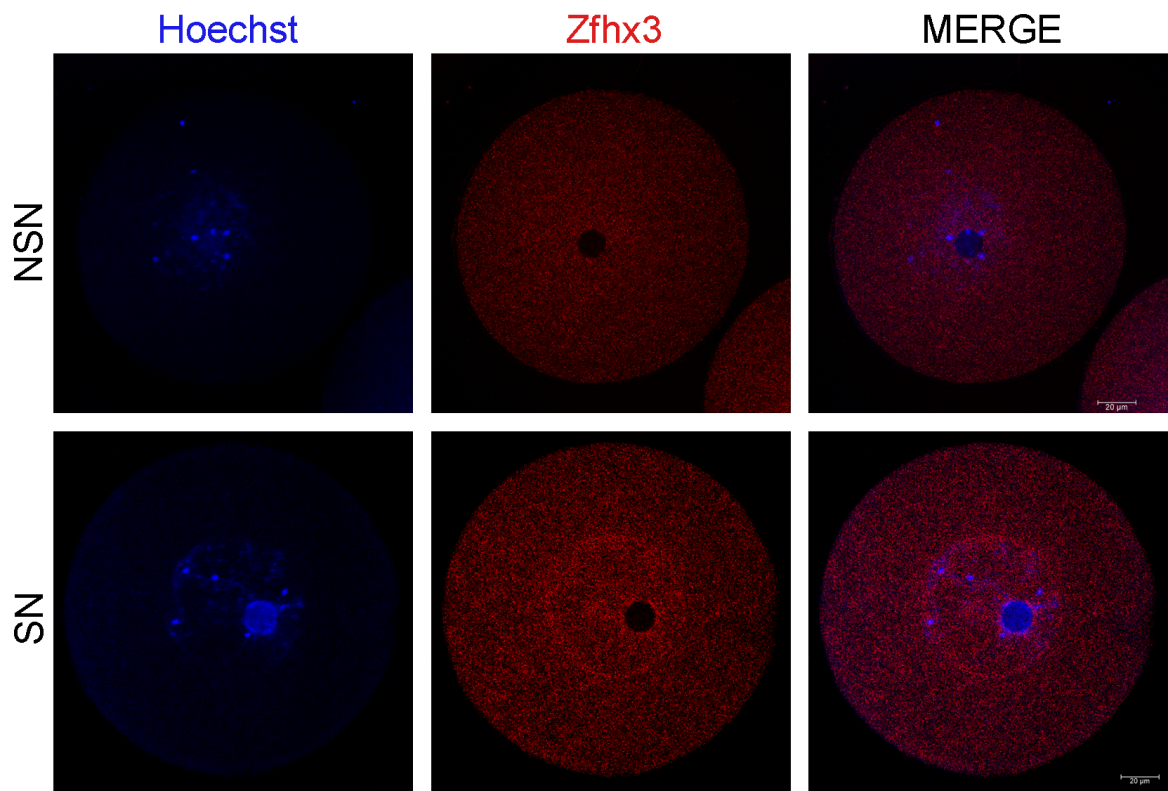
**

**Supplementary Figure 1. Subcellular localizations of Zfhx3 in NSN and SN oocytes.** Immunofluorescence staining and confocal microscopy were used for the detection. Representative images show the subcellular localization of Zfhx3 in NSN (n = 12) and SN (n = 15) oocytes. Scale bar, 20 μm.

# Supplementary Figure 2


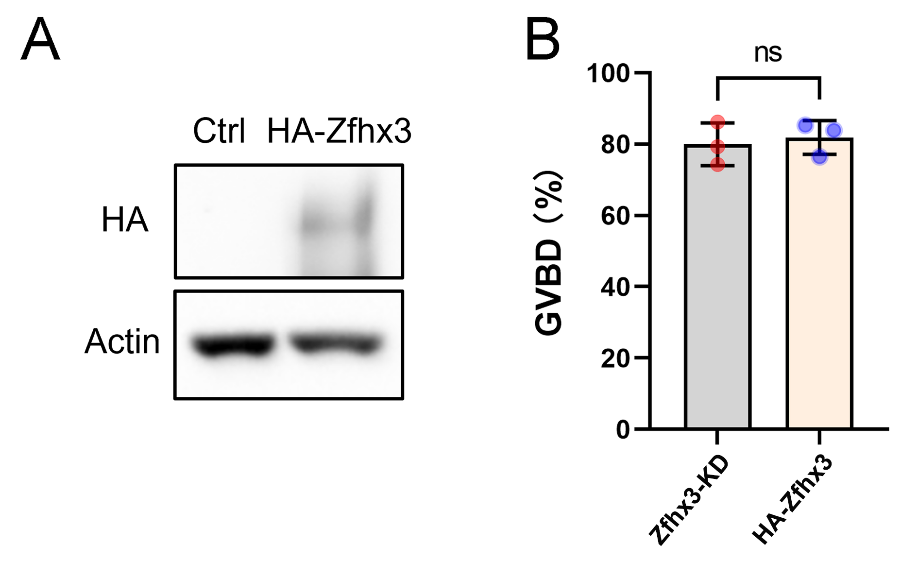


**Supplementary Figure 2.** **Effects of injected HA-*Zfhx3* mRNA on GVBD rates in mouse oocytes.** (A) Detection of HA-Zfhx3 protein expression by western blotting in the control and HA-Zfhx3 groups. (B) A bar graph shows GVBD rates in oocytes from the Zfhx3-KD and HA-Zfhx3 groups. Zfhx3-KD: 79.94 ± 5.99%, n = 93; HA-Zfhx3: 81.88 ± 4.74%, n = 99; *p* > 0.05. Individual dots correspond to independent experiments. The bar height and error bars depict the mean and SD, respectively.
